# Persistence of *Leptospira borgpetersenii* Serovar Hardjo in Refrigerated Raw Milk: A Transmission Risk of Leptospirosis to Humans

**DOI:** 10.3390/pathogens10030291

**Published:** 2021-03-03

**Authors:** Elisa Mazzotta, Letizia Ceglie, Isabella Giurisato, Laura Bellinati, Laura Lucchese, Silvia Marchione, Alda Natale

**Affiliations:** 1 Istituto Zooprofilattico Sperimentale delle Venezie, 35020 Legnaro, Italy; mazzottaelisa@gmail.com (E.M.); lceglie@izsvenezie.it (L.C.); igiurisato@izsvenezie.it (I.G.); lbellinati@izsvenezie.it (L.B.); llucchese@izsvenezie.it (L.L.); smarchione@izsvenezie.it (S.M.); 2 Department of Animal Medicine, Productions and Health (MAPS), University of Padova, 35020 Legnaro, Italy

**Keywords:** leptospirosis, *Leptospira* Hardjo (LH) persistence, bovine milk, raw consumption

## Abstract

*Leptospira borgpetersenii* serovar Hardjo (LH) is an important infectious agent of reproduction pathologies and lactation decline in cattle, with a possible zoonotic role. To figure out the potential zoonotic risk for human raw-milk consumption, the present study aims at assessing the persistence and viability of LH in refrigerated raw milk over a 10-day period, which is set as the maximum time range for raw-milk domestic consumption. A negative sample of fresh raw milk was contaminated with an LH strain (2 × 10^8^ Leptospires/mL) and analyzed by a rrs (16S) gene targeting real-time PCR (rPCR) protocol for LH DNA at days 1, 2, 3, 6, 7, 9, and 10. Seven aliquots of the same sampling time were inoculated into a semisolid EMJH media for bacterial culture. All aliquots tested positive in both rPCR and culture, which demonstrates that raw milk does not alter the detectability and viability of LH, respectively. The analytical sensitivity (LoD, limit of detection) determined for the rPCR (103 Leptospires/mL) was repeatable during the study, whereas it gradually decreased when it came to the bacterial culture. This study demonstrates that bovine raw milk might be a potential vehicle of infection by LH, even when storage conditions are strictly respected.

## 1. Introduction

Leptospirosis is a zoonosis of worldwide distribution, caused by the infection with pathogenic spirochetes of the genus *Leptospira*. The disease is maintained in nature by chronic renal infection of carrier mammals [1]. Typically, humans become infected through the direct exposure to the infected animals or their urine or through indirect contact with contaminated water or soil [2]. In Europe, leptospirosis has been recognized as an emerging infectious disease, in part because of recent large-scale outbreaks associated with recreational activities [3,4]. Leptospirosis is one of the leading global causes of morbidity and mortality [5,6] and has a large impact on public and animal health [7]. Investigations focused on both the spread of the infection and clinical cases have recently been enhanced thanks to the availability of rapid and sensitive diagnostic molecular assays, which are able to provide an early diagnosis in clinical illness and subclinical infections.

There are approximately 300 serovars of *Leptospira* spp. divided into 28 groups [8]. Cattle are recognized as maintenance hosts of Hardjo serovar, causing chronic disease with a subclinical and persistent infection of their reproductive tract [9].

As for some other zoonotic agents, a possible transmission with the bovine raw milk deriving from infected cows is also reported for *Leptospira* [10,11]. Raw milk has frequently been recognized as the source of foodborne illness outbreaks, caused mainly by bacterial agents [12,13,14]. There is considerable public debate on the possible benefits regarding the growing popularity of the consumption of raw milk [15] in light of its positive characteristics, such as nutritional qualities, taste, and health benefits [16]. However, several different pathogens may contaminate raw milk, even when sourced from clinically healthy animals [17]. There are many references available in literature reporting about the detection of *Leptospira* antibodies in bulk milk, performed to investigate the prevalence of *Leptospira* infection or exposure in dairy herd [18,19,20,21,22,23].

Conversely, there are only a few studies that confirm the presence of *Leptospira* spp. in milk [10,24,25], in which the viability of *Leptospira borgpteresenii* serovar Hardjo (LH) in raw milk was found to be low. According to their findings, the authors believe that bovine raw milk can be excluded as a source of Leptospira infection for consumers. Nevertheless, further studies are needed to confirm this assumption [26].

In order to figure out the possible zoonotic risk for human consumption, the present study aims at assessing the potential persistence and survival capability of LH in refrigerated raw milk (Regulation (EU) no. 605/2010) [27] and at the same time wishes to evaluate whether it is able to preserve its viability over a 10 day period. In addition, we established the diagnostic cutoffs of two different analytical methods (biological versus molecular) to be used throughout the study period for detecting the presence of LH.

## 2. Results

As reported in Table 1, aim of the present study was to evaluate the detectability of LH over the entire period of the study, as well as to assess the progressive LH strain dilutions with two different analytical methods (biological versus molecular).

### 2.1. Bacterial Culture Test

**Figure 1:**
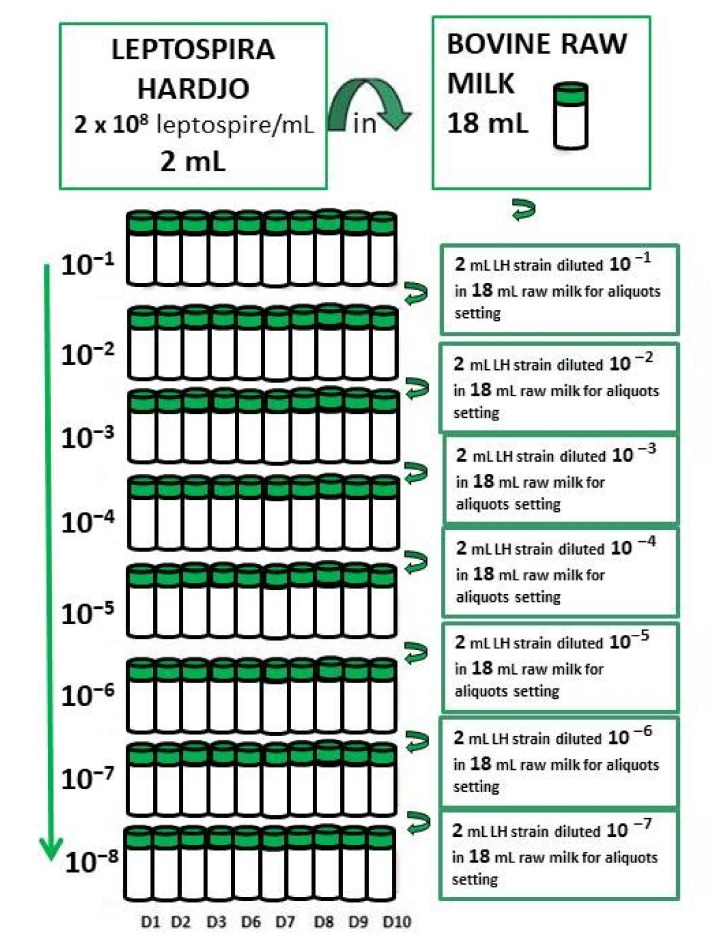
LH culture strain dilutions and aliquots setting for each day of the study period. Milk spiking achieved by inoculating 2 mL of the strain culture at the 2 × 10^8^ LH/mL concentration into 18 mL of raw milk, obtaining a 10^−1^ dilution. Further 10-fold dilutions of the previously spiked milk were set up to 10^−8^. At D1, all the spiked set aliquots were refrigerated at 4 ± 3 °C for sampling until D10. On each day of the study period, one set of aliquots of diluted spiked milk (10^−1^ to 10^−8^) was processed through molecular and biological culture assays.

**Figure 2:**
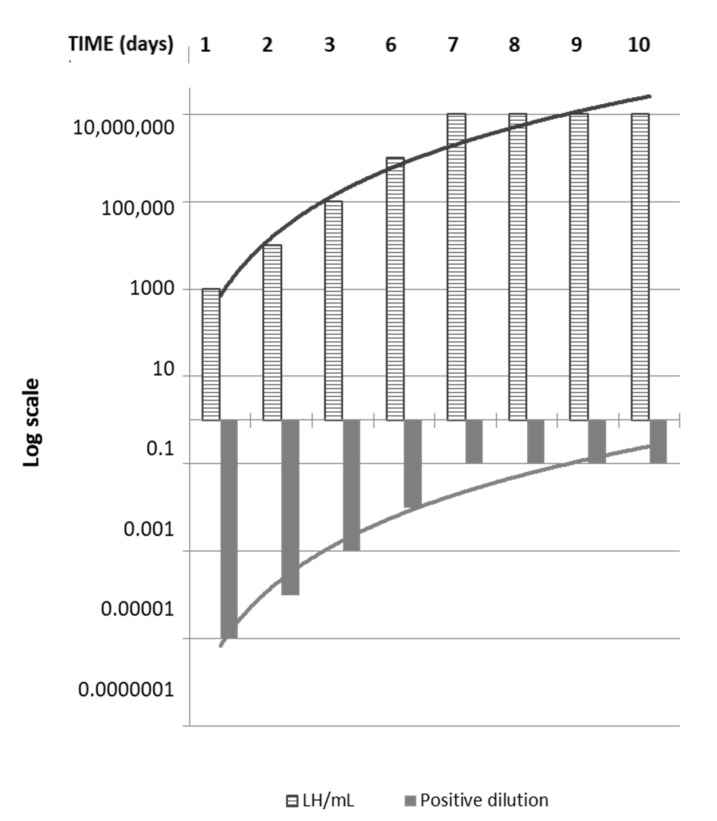
Bacterial culture test results: The results generated by the isolation assay over the study period are represented in the upper part of the graph, where the striped bars (grey diamonds) show the Log [LH] needed by a spiked sample to achieve a positive isolation at each day of observation. Below, the grey bars (light-grey squares) reproduced the Log [LH] of the positive resulted dilutions, showing an expected decreasing trend along with the elapse of time.

From day 1 to day 8 (D1–D8) of the storage period at 4 ± 3 °C, aliquots of the LH spiked milk samples were put in a culture at 30 °C, and they needed about 1 to 5 days of incubation to show a positive bacterial growth (Figure 1). On the other hand, spiked samples inoculated in culture media at D9–10 needed a longer incubation time to show any bacterial growth, which was observed from D8 to D10 (Figure 2).

Sensitivity of the bacterial culture method was assessed. Table 1 reports the highest cutoff value achieved by an LH-positive culture throughout the entire observation period, as well as the rPCR Ct values recorded for each cutoff value (Table 2).

The minimum LH concentration of 10^3^ LH/mL for a positive culture was reached at D1. As expected, the concentration yielding a positive culture increased progressively to the value of 10^7^ LH/mL in samples inoculated from day 7 to day 10 (D7–10). Similarly, the LH viability showed a decreasing trend during the study period, due to both the dilution procedure and to the longer storage time of raw milk (Table 1).

### 2.2. Real-Time PCR Analysis

In all the samples analyzed per time point of the study, the limit of detection (LoD) for the rPCR achieved the same cutoff value as the one observed in the culture assay at D1, corresponding to 10^3^ LH/mL (Table 1 and Table 3).

Conversely, all the spiked samples that tested negative in culture assay during the study period were analyzed with the same rPCR method and showed some PCR positivity.

**Figure 3:**
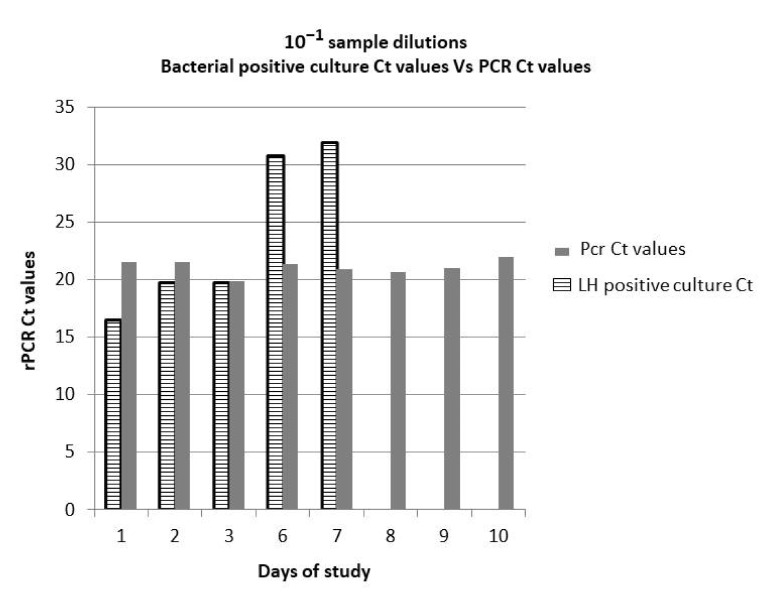
The plot shows the trend of Ct values recorded by molecular analysis in positive bacterial culture (striped bars) and LH spiked samples analyzed by rPCR (grey bars) for 10^−1^ sample dilution, recorded during the study period.

Overall, in this study we observed a progressive increase of Ct in the spiked culture samples proportional to the milk storage period. In particular, by comparing Ct data recorded in 10^−1^ dilutions of positive bacterial culture with those obtained in all the LH spiked samples analyzed by rPCR, an increase of Ct values was observed only in bacterial culture samples (Figure 3).

**Figure 4:**
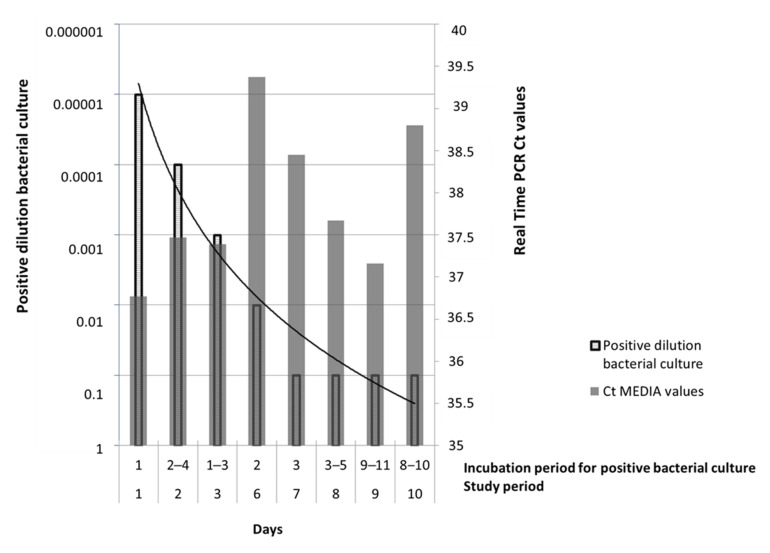
The plot shows the trend of data obtained in isolation and molecular analysis: bacterial cultures (striped bars) (Table 1, D1 to D10) and no contaminations occurred during the storage time (4 °C storage temperature). The negative raw-milk controls collected at D1 and D10 of the study were analyzed with rPCR and tested negative, as expected. Similarly, no bacterial growth was observed in the two bacterial cultures (D1 and D10), which means that the raw-milk samples used in the study were free from environmental *Leptospira* and that no further contaminations occurred during storage time (4 °C storage temperature).

By comparing the Ct values obtained with the molecular and biological methods, we observed that the sensitivity of the bacterial culture test was comparable to the one obtained in rPCR only on the first day (D1) of the study (corresponding to 10^3^ LH/mL); at D2–D3 and D6 sensitivity decreased following a logarithmic scale until it reached a constant Ct value (10^−1^) lasting from D7 to D10. Conversely, when performing the rPCR assay results of all the samples analyzed remained unchanged per each time study point (10^3^ LH/mL, particularly LoD = 10^−5^) (Figure 4).

## 3. Discussion

In order to figure out the potential zoonotic risk for human consumption, our aim was to assess the potential persistence and survival capability of LH in refrigerated raw milk and to evaluate its capability to preserve the viability of the bacterial agent over a 10-day period. In addition, we wanted to measure the diagnostic sensitivity for two different analytical methods (biological versus molecular) to identify the presence of LH over the entire study period. Despite the possible anti-*leptospira* inhibiting factors [28,29] and the storage temperature which was uncomfortable for the bacterial growth and its survival, the presence of LH was reported over the entire study period (10 days at storage temperature 4 ± 3 °C). The bacterial culture and isolation were particularly employed to evaluate the viability of the leptospires; we observed a logarithmic reduction in bacterial replication capability proportional to the increase of storage time (4 °C) and samples dilution (Figure 4), reaching a plateau at D7 (10^−1^, 10^3^ LH/mL). This event may be explained by the lowering in viable bacterial load due to the storage period and to the characteristic bovine raw-milk antibacterial factors [28,29]. However, these conditions did not fully reduce leptospires viability; in fact, all the cultures were found positive despite the increasing incubation period. These aspects demonstrate that bovine raw milk may represent a zoonotic biohazard for human consumption. Both analytical methods verified the detectability of *Leptospira* in refrigerated raw milk. The presence of bacterial DNA was reported throughout the study period (10 days at storage temperature 4 ± 3 °C): this aspect highlighted the highest sensitivity of rPCR assay and showed reproducible results for all the samples analyzed per each time point of study (10^3^ LH/mL, particularly LoD = 10^−5^).

LH infection in cows usually shows nonsuggestive symptoms, and infected subjects often seem to be clinically healthy. Clinical manifestations could be a uterus infection and abortion in pregnant cows, premature, stillbirth calves, or even healthy calves which could be infected by congenital leptospirosis (leptospiruria) [30]. The presence of LH in infected kidneys was reported for a period of 542 days [31]; as a matter of fact, the cows developed a chronic interstitial nephritis, and they could potentially spread LH for a prolonged and intermittent period. In addition, LH infection in cows could cause atypical mastitis, leading to a marked decrease in milk production with a morphological alteration of the mammary gland (soft consistence, pale skin, and dense milk) for a period ranging from 2–10 days [24]. This clinical manifestation could be the only clinical sign, and it might be mistaken for mastitis due to the presence of other bacteria (*Escherichia coli* or *Pasteurella multicida*). In fact, it is well known that the routine microbiological test applied for the diagnosis of mastitis fail to detect *Leptospira*. Therefore, during routine practice, it could be difficult to identify lactating animals infected by LH, although the excretion of LH in infected cow’s milk has been reported [10,32] and it may represent a zoonotic risk for human health [2,9,33,34]. Both *Leptospira* DNA and viable LH were detected throughout the study period, which demonstrates that LH could be isolated in infected raw milk stored at +4 °C for 10 days (conventional expiring period and storing conditions for raw milk), even if with a decreasing replication capability and viability from days D2–D6 and D7–D10, respectively. This condition may constitute a biohazard and a zoonotic risk for human consumption. Antimicrobial and thermic treatments (e.g., pasteurization) applied in food production industries can ensure the complete elimination of *Leptospira*.

Foodborne disease accounts for substantial morbidity and mortality in different countries, and raw milk has frequently been identified as the source of foodborne illness outbreaks [12,13,14,35,36]. Regulation (EU) no. 605/2010 of the commission of 2 July 2010, establishes, “animal and public health and veterinary certification conditions for the introduction into the European Union of raw milk and dairy products intended for human consumption” (OJ L 175 of 10 July 2010, pp. 1–24) [27]. In truth, neither the OIE Manual of World Organization for Animal Health 2018 [37], nor Regulation (EU) 2017/625 of the European Parliament and of the Council (on official controls and other official activities performed to ensure the application of food and feed law, rules on animal health and welfare, and plant health and plant protection products) [38] and Regulation (EU) 2016/429 of the European Parliament and of the Council of 9 March 2016, (on transmissible animal diseases and amending and repealing certain acts in the area of animal health “Animal Health Law”) establish any specific surveillance and monitoring programs for leptospirosis in cattle [39]. Because of the difficulties in diagnosing LH in zootechnical practice, it is recommended to implement sero-surveillance programs for the dairy farm system through serological kits, such as ELISA-Ag specific for LH. These tests are easily available, non-invasive, and low cost; in particular, they could be performed on individual serum sample or on bulk milk.

## 4. Materials and Methods

### 4.1. Bacterial Strain and Culture Media

*Leptospira borgpetersenii* serogroup Sejroe serovar Hardjo type Hardjobovis (LH) (strain IZSLER L224 is distributed by the Italian Reference Center for Animal Leptospirosis to be used as antigen in the routine diagnostic micro-agglutination test) is usually maintained in a liquid EMJH medium prepared following Johnson et al. [40], added with the enrichment solution DIFCO (Becton Dickinson Italia S.p.A., Milan), and incubated at 30 ± 2 °C for 7–10 days with weekly passages in a new medium. To carry out the experimental contaminations, EMJH liquid and semisolid culture media were used, according to previous studies [40], with the addition of DIFCO enrichment solution (Becton Dickinson Italia S.p.A, Milan, Italy). In order to perform the spiking procedure, the LH strain was grown for approximately 96 h to reach the concentration of 2 × 10^8^ bacterial/mL, as requested for the micro-agglutination test [37,41].

After the incubation period, the culture was examined by the dark-field optic microscope to evaluate bacterial viability. The concentration of LH has been estimated by means of a spectrophotometer, reading at 400 nm, and obtaining a transmittance (%T) value between 60% and 70% [37,41]. Early in the morning on the first day of the study, a bovine raw-milk sample was collected from a milk consumer automatized dispenser. The sample was stored at 4 ± 3 °C, in order to maintain the specific chemical-physical and microbiological characteristics unaltered. The raw-milk sample was analyzed by means of rPCR to ensure the absence of *Leptospira* spp. contamination.

Milk spiking was achieved by inoculating 2 mL of the strain culture at the 2 × 10^8^ LH/mL concentration (%T 63) into 18 mL of raw milk, obtaining a 10^−1^ dilution. On the first day (D1), further 10-fold dilutions of the previously spiked milk sample were subsequently prepared up to 10^−8^, corresponding to 100 units of LH/mL (Figure 1). Similarly, on the same day (D1), all the spiked aliquots were stored and refrigerated at 4 ± 3 °C for successive sampling until the end of the study period (D10). For the entire period of the study (D1–D10), one set of aliquots of diluted spiked milk (10^−8^ to 10^−1^ LH/mL), i.e., two aliquots of 1 mL each, were daily processed to attempt both isolation and rPCR detection. The molecular protocol and the isolation assay were performed on the same sample sets, in order to establish the analytical sensitivity of the two methods. In order to do so, for each dilution (10^−8^ to 10^−1^ LH/mL) two aliquots of 1 mL each were collected throughout the study period, to be daily processed for isolation attempts and for rPCR detection, respectively.

To evaluate the potential zoonotic risk of LH in raw milk and its survival in this matrix, all samples were refrigerated at 4 ± 3 °C, simulating the storing conditions of milk for human food consumption.

In the same way, aliquots of raw milk not inoculated and used as negative controls, were stored at the same conditions and checked over the entire study period to ensure the negativity of the originally collected sample.

### 4.2. Raw Milk Samples Spiking

At days 1, 2, 3, 6, 7, 8, 9, and 10 of the study period, 1 mL from each of the 8 dilutions (from 10^−1^ to 10^−8^) of LH contaminated raw milk was inoculated in a culture media (Figure 1). At D1 and D10, 1 mL of raw milk from the original sample was inoculated in the culture media as a control sample. All samples were filtered with a 0.45 nm filter before the inoculation in the EMJH culture medium, both liquid and semisolid, to avoid the contamination by other microorganisms [2,41,42]. According to the isolation procedure, for each sample, two dilutions (10^−2^ and 10^−3^) were prepared. The bacterial cultures were incubated at 30 ± 2 °C for 26 weeks in order to assess the negativity of the original sample [37] (Figure 1).

### 4.3. Laboratory Analyses

#### 4.3.1. Culture Evaluation

From the first to the twenty-first day, a 5 µL aliquot of each culture was daily examined by the dark-field optic microscope, in order to assess the growth and the viability of LH in the culture medium through the increasing dilutions of bovine refrigerated raw milk. This assessment was performed on a weekly basis. The examination was performed on a fresh sample, using a focus 100× to a general observation, and a focus 320× to evaluate individual *Leptospira* in case of low culture growth. The minimum concentration required to identify one *Leptospira* for each microscopic optic field is of 10^4^ LH/mL [43].The culture was considered positive if one viable *Leptospira* was detected; conversely, the culture was considered negative if no viable *Leptospira* were observed at the end of the 26 weeks of incubation, according to the OIE procedure [37]. In case of contamination by other microorganisms, the cultures were filtrated through a 0.22 μm sterile syringe filter and sub-cultured in fresh EMJH media. All the results were analyzed to define the sensitivity of the bacterial culture method used to evaluate the presence of LH in D1 spiked milk dilutions (10^−1^ to 10^−8^) (limit of detection, LoD). Throughout the study period, the survival curve of each positive culture sample was outlined.

#### 4.3.2. Molecular Investigation

##### Sample Setting and DNA Extraction

Each day (1, 2, 3, 6, 7, 8, 9, 10) of the study period, 1 mL aliquot of the raw contaminated milk (dilution from 10^−1^ to 10^−8^) was centrifuged at 12.000× *g* rpm for 20 min. Supernatants were discarded and pellets were suspended in 200 µL of sterile PBS (Phosphate buffered saline), after addition of 10 µg of Poly A, in order to improve the rescue and detection rate of the investigated genetic material. All aliquots were processed for DNA extraction and analysed, as well as two negative controls consisting of raw milk original samples collected at D1 and D10. Furthermore, 2 mL aliquots of EHJM liquid medium cultures at 10^−1^ dilution referred to days 7 and 21 were examined with the same procedure. DNA extraction was performed by means of the High Pure PCR Template Preparation Kit^®^ (Roche Diagnostics GmbH, Mannheim, Germany), according to the manufacturer’s instructions (paragraph Isolation of nucleic acids from Bacteria or Yeast). The incubation steps with lysozyme and proteinase K. DNAs were stored at −20 °C, until the amplification step.

##### Qualitative Real-Time PCR Analyses

*Leptospira* bacterial DNA was submitted to a TaqMan-based real-time PCR protocol targeting an 87 bp fragment of the rrs gene encoding the 16S rDNA, expressed in vivo by leptospires, modified by Smythe et al. (2002) [44].

Briefly, rPCR was carried out in a 25 µL final volume, containing 1 µL of extracted DNA, 12.5 µL of 2× Master Mix TaqMan Universal (Thermo Fisher Scientific, Life Technologies LTD, Warrington, UK), 1.5 µL of each primer (final concentration 300 nm), Primer LEPTO F (5′ CCC GCG TCC GAT TAG 3′) and Primer LEPTO R (5′ TCC ATT GTG GCC GRA CAC 3′), and 1.5 µL of FAM–CTC ACC AAG GCG ACG ATC GGT AGC 3′-TAMRA probe (final concentration 100 nm), and 6 µL of nuclease-free water.

Each amplification assay included a negative control (nuclease-free water), a negative bacterial genomic control (DNA of *Leptospira biflexa* serovar Patoc), and a positive control (DNA of L. interrogans serovar Icterohaemorrhagiae), and each sample was tested in duplicate. Real-time PCR was performed on a 7900HT Fast Real-time PCR System (Thermo Fisher Scientific) under the following thermal conditions: a hot-start step at 50 °C for 2 min, a holding step at 95 °C for 10 min, and 45 cycles of 95 °C for 15 s and 60 °C for 60 s. Samples with Ct < 40 after amplification were considered positive, whereas samples having no FAM fluorescence signal or with Ct ≥40 were considered negative. The analytical sensitivity of this protocol (limit of detection, LoD) was previously determined by using serial dilutions starting from a culture of the reference strain at a concentration of 2 × 10^8^ LH/mL, estimated by the transmittance value (%T) with a spectrophotometer, ranging from 60–70% [OIE reference Manual, 2018] [37].

## 5. Conclusions

The consumption of raw milk is widespread on a global scale and may vary according to different cultural and social environments. Foodborne diseases account for substantial morbidity and mortality, and raw milk has frequently been identified as the source of foodborne illness outbreaks. However, thermic treatments (e.g., pasteurization) and other antimicrobial treatments applied in food production industries can ensure the complete elimination of *Leptospira*. This study demonstrates the potential zoonotic risk that human consumption of raw milk may have and shows that LH can be isolated in infected raw milk stored at 4 °C within a 10-day period (conventional expiring period and storing conditions for raw milk). In addition, we observed that the bacterial culture test sensitivity is comparable to the one measured for rPCR only on the first day (D1) of the study (10^−5^, 10^3^ LH/mL). Conversely, the sensitivity of the assay when performed by rPCR showed unchanging results for all the analyzed samples per each time point of the study.

## Figures and Tables

**Table 1:** Schematic description of the study period (D1–10); the Leptospira borgpetersenii serovar Hardjo (LH) strain dilution in raw milk; the Ct values detected via rPCR assay (Section A); and the days to obtain a bacterial cultural test positivity (Section B).

**Section A—rPCR Results (Ct Values)**								
**LH Strain Dilution**	**D1**	**D2**	**D3**	**D6**	**D7**	**D8**	**D9**	**D10**
10^−1^	21.53	21.54	19.87	21.35	20.91	20.64	21.01	21.94
10^−2^	23.96	23.64	24.78	25.14	24.93	25.05	25.49	25.96
10^−3^	27.89	28.16	28.56	29.07	29.95	29.55	29.81	28.96
10^−4^	31.25	41.38	32.78	33.09	34.93	33.13	33.05	32.09
10^−5^	36.77	37.47	37.39	39.37	38.45	37.67	37.16	38.80
10^−6^	Undet.	Undet.	Undet.	Undet.	Undet.	Undet.	Undet.	Undet.
10^−7^	Undet.	Undet.	Undet.	Undet.	Undet.	Undet.	Undet.	Undet.
10^−8^	Undet.	Undet.	Undet.	Undet.	Undet.	Undet.	Undet.	Undet.
**Section B—Culture Test Results**								
**LH Strain Dilution**	**D1 (d)**	**D2 (d)**	**D3 (d)**	**D6 (d)**	**D7 (d)**	**D8 (d)**	**D9 (d)**	**D10 (d)**
10^−1^	+(1)	+(2)	+(2)	+(2)	+(3)	+(5)	+(9)	+(10)
10^−2^	+(5)	+(5)	+(6)	+(8)	Undet.	Undet.	Undet.	Undet.
10^−3^	+(5)	+(6)	+(10)	Undet.	Undet.	Undet.	Undet.	Undet.
10^−4^	+(7)	+(15)	Undet.	Undet.	Undet.	Undet.	Undet.	Undet.
10^−5^	+(12)	Undet.	Undet.	Undet.	Undet.	Undet.	Undet.	Undet.
10^−6^	Undet.	Undet.	Undet.	Undet.	Undet.	Undet.	Undet.	Undet.
10^−7^	Undet.	Undet.	Undet.	Undet.	Undet.	Undet.	Undet.	Undet.
10^−8^	Undet.	Undet.	Undet.	Undet.	Undet.	Undet.	Undet.	Undet.

Undet. = D = day of inoculum; (d) = n. of days to show a LH growth in culture.

**Table 2:** Bacterial culture test diagnostic cutoffs.

Observation Period (Days)	Incubation Period to Obtain a Positive Culture Isolation (Days)	LH/mL	rPCR Ct Values Recorded in the Corresponding Positive LH Culture Over the Observation Period
D1	1	10^3^	36.77
D2	2–4	10^4^	32.52
D3	1–3	10^5^	28.56
D6	2	10^6^	25.14
D7	3	10^7^	20.91
D8	3–5	10^7^	20.63
D9	9–11	10^7^	21.01
D10	8–10	10^7^	21.94

**Table 3:** Limit of detection (LoD) of real-time PCR (rPCR) per time point: ranges of values, Ct media, and LH concentration, respectively.

Day of Study	Ct Values (Threshold = 40)	Ct MEDIA Values	Sample Dilution	LH/mL
D1	36.74–36.80	36.77	10^−5^	10^3^
D2	38.31–36.63	37.47	10^−5^	10^3^
D3	36.41–38.37	37.39	10^−5^	10^3^
D6	39.18–39.57	39.37	10^−5^	10^3^
D7	38.37–38.53	38.45	10^−5^	10^3^
D8	37.56–37.78	37.67	10^−5^	10^3^
D9	38.16–36.16	37.16	10^−5^	10^3^
D10	38.80—Undetec.	38.80	10^−5^	10^3^

Undetec. = undetectable.

## Data Availability

The data presented in this study are available in article.
